# Using 3D Heart Printing for Planning to Repair a Complex Congenital Heart Disease via Minimal Invasive Thoracotomy

**DOI:** 10.1016/j.jaccas.2025.104200

**Published:** 2025-06-25

**Authors:** Mohammed Abutaqa, Hamza A. Abdul-Hafez, Hasan Alkhatib, Helmi Mahmoud Tamimi, Bilal Adwan

**Affiliations:** a Pediatric Cardiology Department, Palestinian Medical Complex, Ramallah, Palestine; b Faculty of Medicine and Health Sciences, Department of Medicine, An-Najah National University, Nablus, Palestine; c Department of Cardiac Surgery, Al Makassed Hospital, Jerusalem, Palestine; d Department of Cardiology, Al Makassed Hospital, Jerusalem, Palestine

**Keywords:** 3D heart printing, congenital heart disease, cor triatriatum sinister, double superior vena cava, minimally invasive cardiac surgery

## Abstract

**Background:**

Cor triatriatum sinister is a rare congenital heart disease that can mimic left atrial obstructive lesions. Symptoms vary based on the severity of obstruction.

**Case Summary:**

We report a case of a 14-year-old girl with severe exercise intolerance. Echocardiography and computed tomography scan revealed a fibromuscular membrane causing left atrial obstruction and an anomalous venous drainage with a persistent left superior vena cava to the coronary sinus. Surgical intervention is definitive, typically via sternotomy.

**Discussion:**

Given the complexity and lack of expertise in minimal invasive thoracotomy for such lesions, we used 3-dimensional (3D) heart printing for virtual surgical planning. This approach facilitated a safe and precise repair. To our knowledge, this is the first reported case using 3D printing to enable a minimally invasive thoracotomy for cor triatriatum sinister correction.

**Take-Home Messages:**

Minimally invasive approaches should not be dismissed for complex congenital heart defects. Multimodal imaging can enhance surgical feasibility.

## Visual Summary

**Figure:**
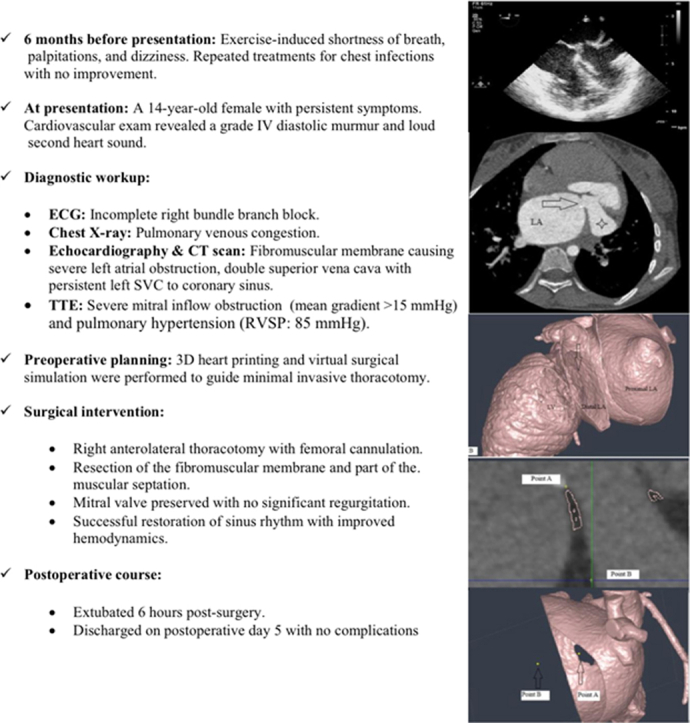

**Visual Summary:**
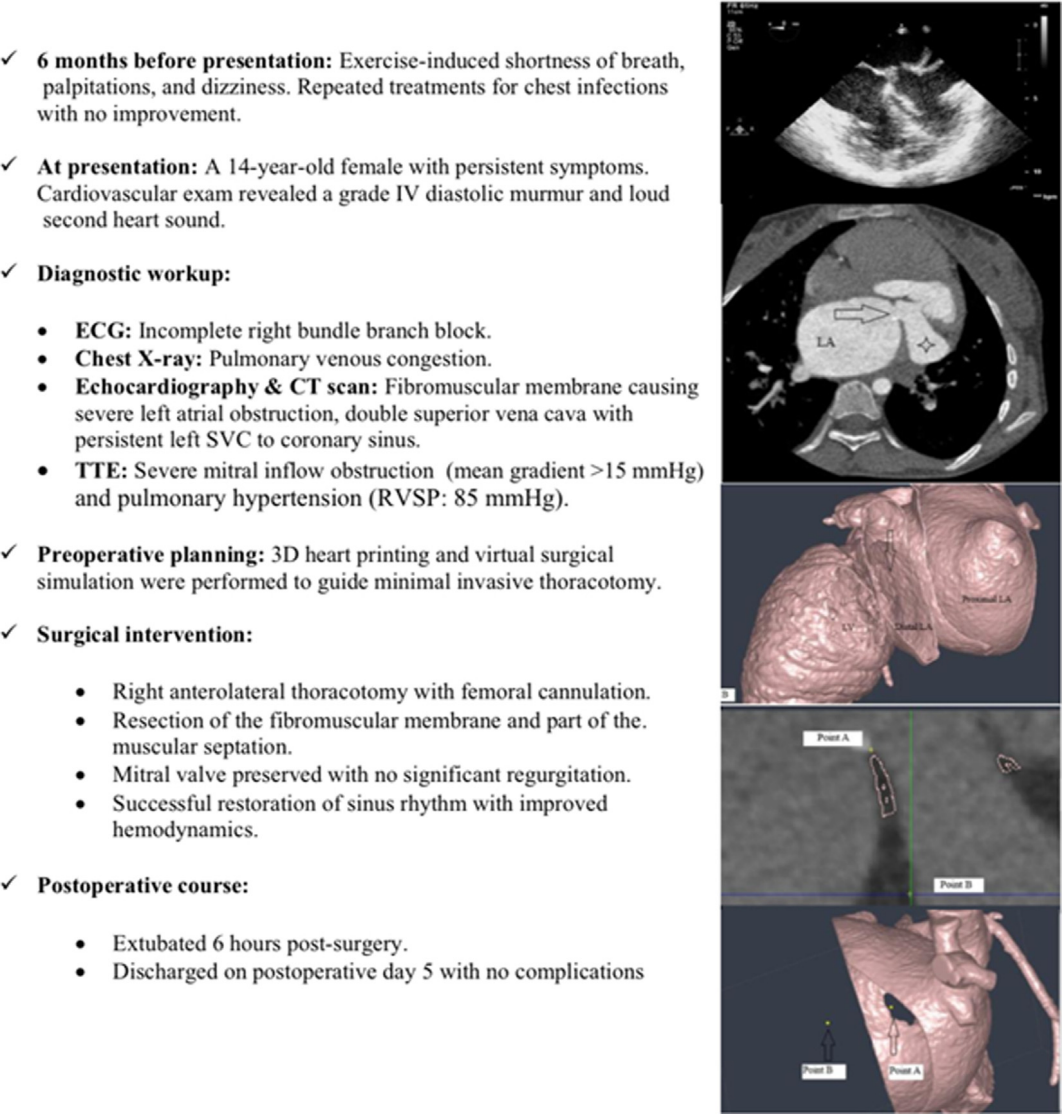
From Presentation to Recovery

Cor triatriatum is a rare congenital cardiac anomaly characterized by an abnormal fibromuscular membrane within the atrium, dividing it into 2 champers and causing inflow obstruction to the respective ventricles.^1^ This condition can involve the left atrium, which is called cor triatriatum sinister (CTS), or the right atrium, known as cor triatriatum dexter.^1^^,^^2^ Typically, the 2 chambers are connected by ≥1 openings of varying size and number.^3^

**Take-Home Messages**

• Minimally invasive thoracotomy can be a safe and effective approach for repairing complex congenital heart defects, offering faster recovery, less pain, and minimal surgical scarring compared with traditional sternotomy.
• 3D heart printing serves as a valuable tool in planning and executing challenging cardiac surgeries, allowing surgeons to perform virtual simulations and optimize surgical strategies before the actual procedure.
• Multimodality imaging, including echocardiography, CT, and 3D printing, enhances surgical precision by providing a detailed anatomic roadmap, reducing intraoperative risks, and improving patient outcomes.
• Complex cardiac anomalies should not be a contraindication for minimally invasive techniques. With proper preoperative planning using advanced imaging and 3D modeling, even rare congenital heart diseases can be successfully treated via less invasive approaches.

The incidence of cor triatriatum ranges from 0.1% to 0.4% of all congenital heart disorders.^1^^,^^4^ CTS is often associated with other congenital anomalies, including atrial septal defect, patent foramen ovale, persistent left superior vena cava (SVC), mitral valve regurgitation, isolated pulmonary artery stenosis, and tetralogy of Fallot.^5^^,^^6^

Clinically, CTS usually mimics mitral valve stenosis, presenting with symptoms such as shortness of breath, palpitation, hemoptysis, transient ischemic attack, syncope, right heart failure, and hypoxemia.^7^^,^^8^ However, the age of presentation varies widely, ranging from infancy to adulthood, depending on the presence of associated atrial septal defect and the size of the connecting fenestrations.

Using advance imaging modalities, including computed tomography angiography, magnetic resonance imaging, and echocardiography, is essential for confirming the diagnosis and optimizing the management and outcomes. Three-dimensional echocardiography, with its enhanced spatial orientation, provides detailed visualization of the membrane's fenestrations and anatomic features, enabling better surgical planning.^9^ However, surgical correction, including atriotomy, excision of the interatrial membrane, and repair of associated defects, is the definitive treatment, with a favorable long-term survival rate of 80% to 90%.^10^

Here, we present a rare case of a 14-year-old girl who presented with dyspnea, palpitations, and dizziness. Using multiple imaging modalities, she was diagnosed with complex CTS accompanied by double SVC and significant mitral inflow obstruction. Sternotomy is the usual surgical approach, and minimal invasive thoracotomy has rarely been used, if ever, to correct such a complex lesion. We used a 3-dimensional (3D) heart printing model to perform a virtual minimally invasive thoracotomy surgical approach to correct the CTS, so the surgeon could become oriented. The patient underwent successful minimally invasive surgery via anterior thoracotomy, resulting in excellent postoperative outcomes.

## History of Presentation

A 14-year-old girl presented to our hospital with a 6-month history of exercise-induced shortness of breath, accompanied by palpitations and recurrent dizziness. However, she had been treated multiple times for chest infections over the past months without significant improvement. Her medical, surgical, and family history were unremarkable. She denied symptoms of fever, hemoptysis, syncope, orthopnea, and headache.

## Diagnostic Work-Up

On physical examination, her vital signs were stable. Cardiovascular examination revealed a grade IV diastolic murmur best heard over the mitral area and a loud second heart sound. Other systemic examinations, including respiratory, neurologic, and gastrointestinal assessments, were unremarkable.

**Figure 1:**
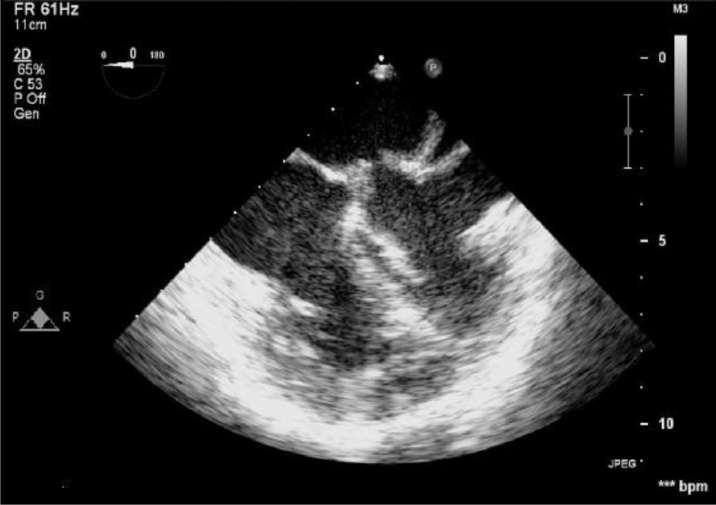
Transesophageal Echocardiography Image Four-chamber view shows the fibromuscular membrane causing septation of the left atrium.

**Figure 2:**
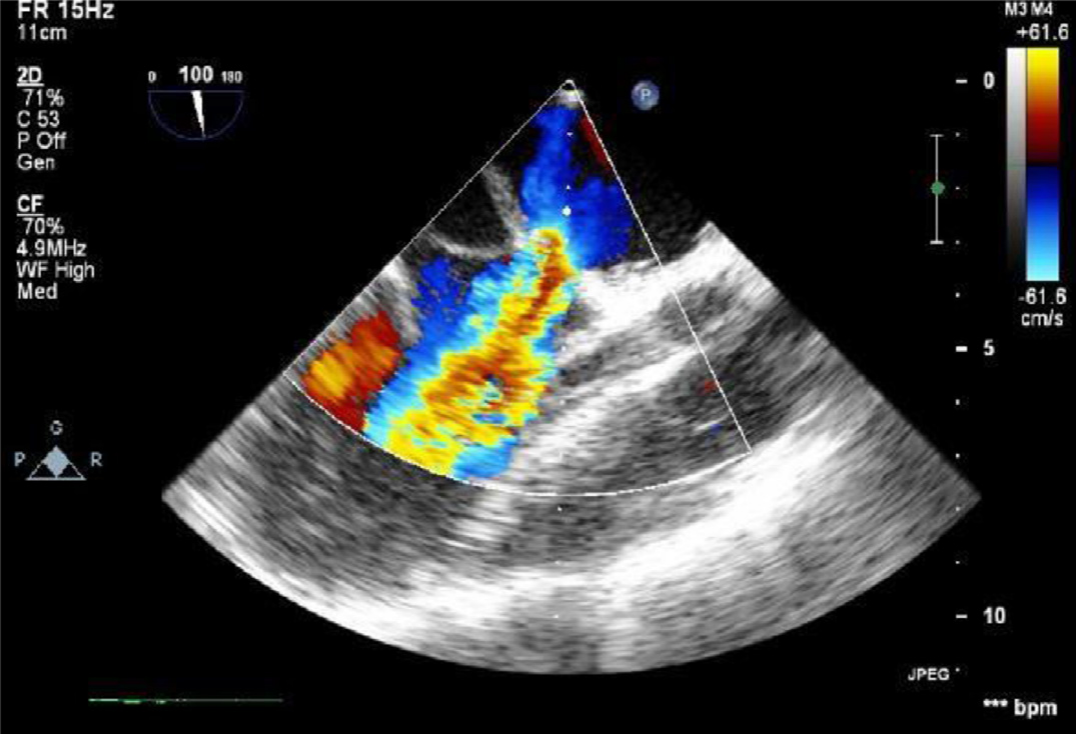
Transesophageal Echocardiography Image Long-axis view shows the turbulent flow in diastole across the opening in the fibromuscular membrane left between the proximal and distal left atrial cavities.

**Figure 3:**
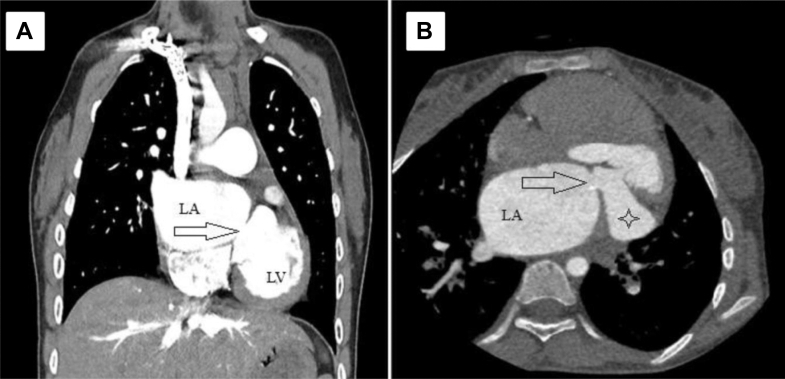
CT Scan of Cor-Triatriatum (A and B) Computed tomography scan images with arrows point to the 8- × 9-mm opening in the fibromuscular membrane that separates the LA into 2 cavities. The star is locating the LA appendage. LA = left atrium; LV = left ventricle.

Electrocardiography demonstrated an incomplete right bundle branch block. Chest radiograph showed congested lungs with prominent perihilar markings indicating pulmonary venous congestion. For further evaluation, transthoracic echocardiography and transesophageal echocardiography were done and showed a fibromuscular membrane separating the left atrium with severe obstruction (Figures 1 and 2). Computed tomography scan of the chest was performed, revealing a septation within the left atrium (Figure 3). Additionally, it also identified a double SVC with a persistent left SVC draining into the coronary sinus.

**Figure 4:**
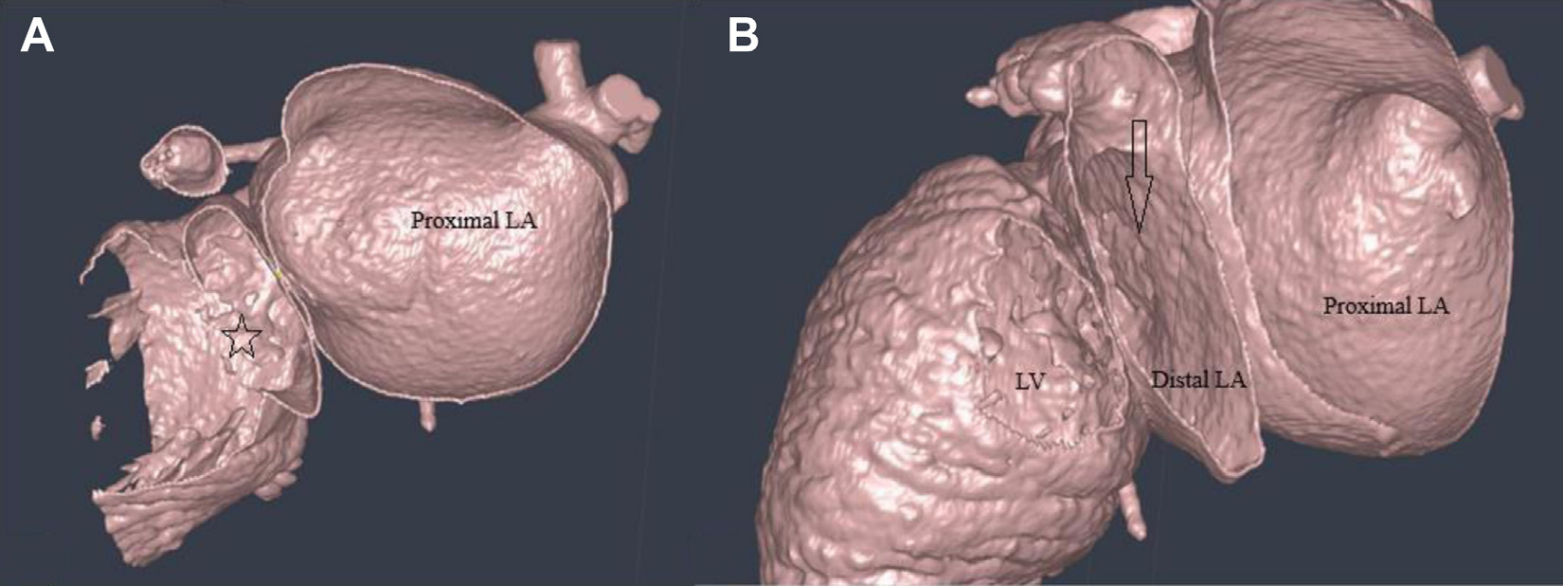
Images of the 3-Dimensional Printed Model We can see the dilated proximal LA separated by the fibromuscular membrane from the distal LA (A). The star is located over the leaflets of the mitral valve in opening position. The arrow in B points to the opening between the 2 left atrial cavities. Abbreviations as in Figure 3.

**Figure 5:**
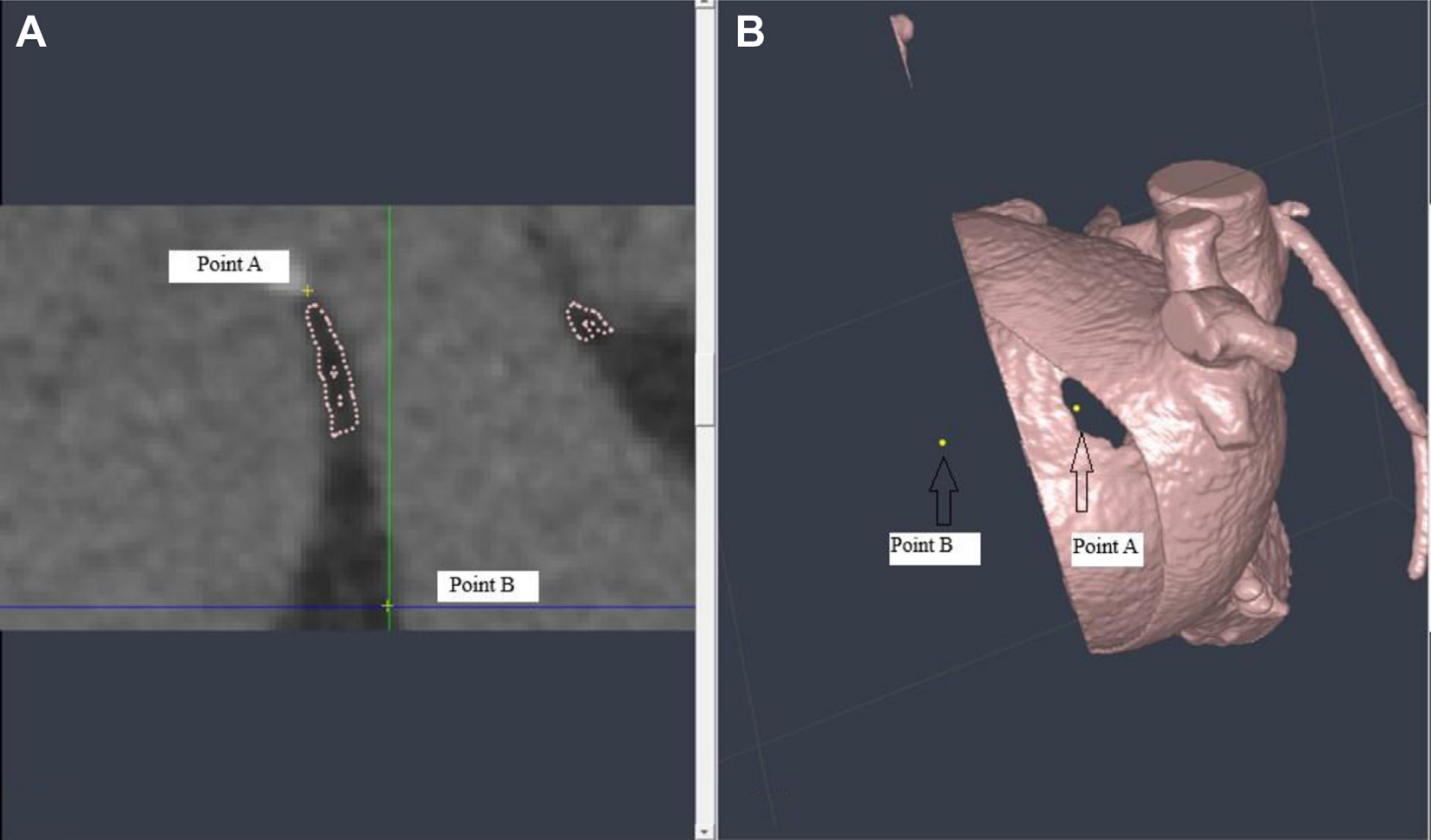
Combining CT Views and 3D Printing Model to Plan Surgery Combining the computed tomography scan image (A) with the 3-dimensional model image (B) to plan for surgery. It was decided to enlarge the opening to a circle with a diameter extending from point A to point B; therefore, we can open the obstruction and keep other structures away from injury. The distance between A and B is approximately 2.5 cm.

Hemodynamic assessment via transthoracic echocardiography revealed a significantly elevated mean pressure gradient (>15 mm Hg) across the membrane, indicating severe obstruction of mitral inflow. This obstruction was associated with severe pulmonary hypertension, with an estimated right ventricular systolic pressure of 85 mm Hg. Anomalous venous drainage, specifically a persistent left SVC, was also confirmed. Given the hemodynamic severity of the mitral outflow obstruction and associated pulmonary hypertension, the patient was admitted for surgical intervention. Based on the CT scan images, we performed a complete segmentation of the patient's heart and 3D printing using the Segment 3D-Print program (Figure 4). The surgeon was then allowed to perform virtually slicing of the model while using combined images with the CT scan (Figure 5), so he knew the direction of resection away from the coronary sinus and the mitral valve leaflets (very close to the membrane) and the diameter of the circle he would need to create. The presence of the anomalous drainage of the left SVC, dilating the coronary sinus, was a challenge.

## Interventions and Outcome

**Figure 6:**
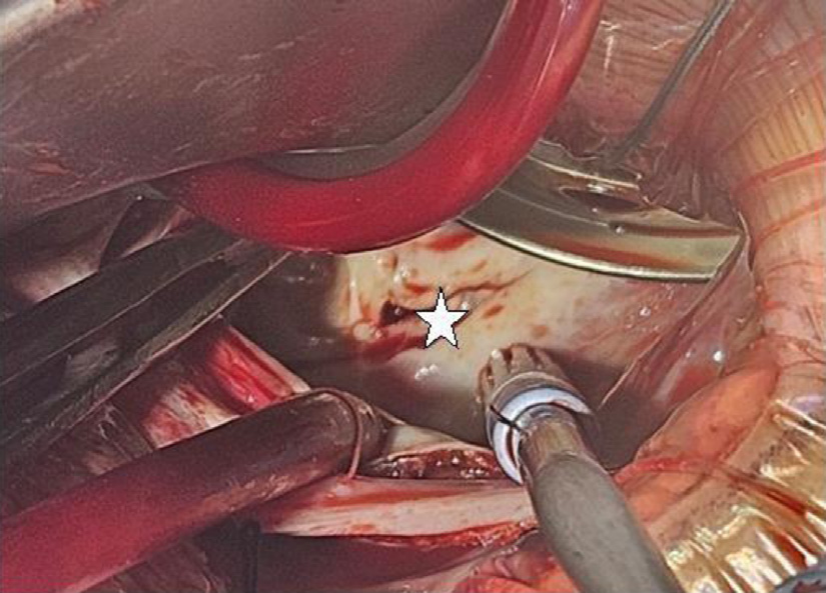
Intra-Operative View of Cor-Triatriatum Surgical view via thoracotomy showing the opening (star) between the proximal and distal parts of the left atrium. Part of the mitral valve is seen through it.

**Figure 7:**
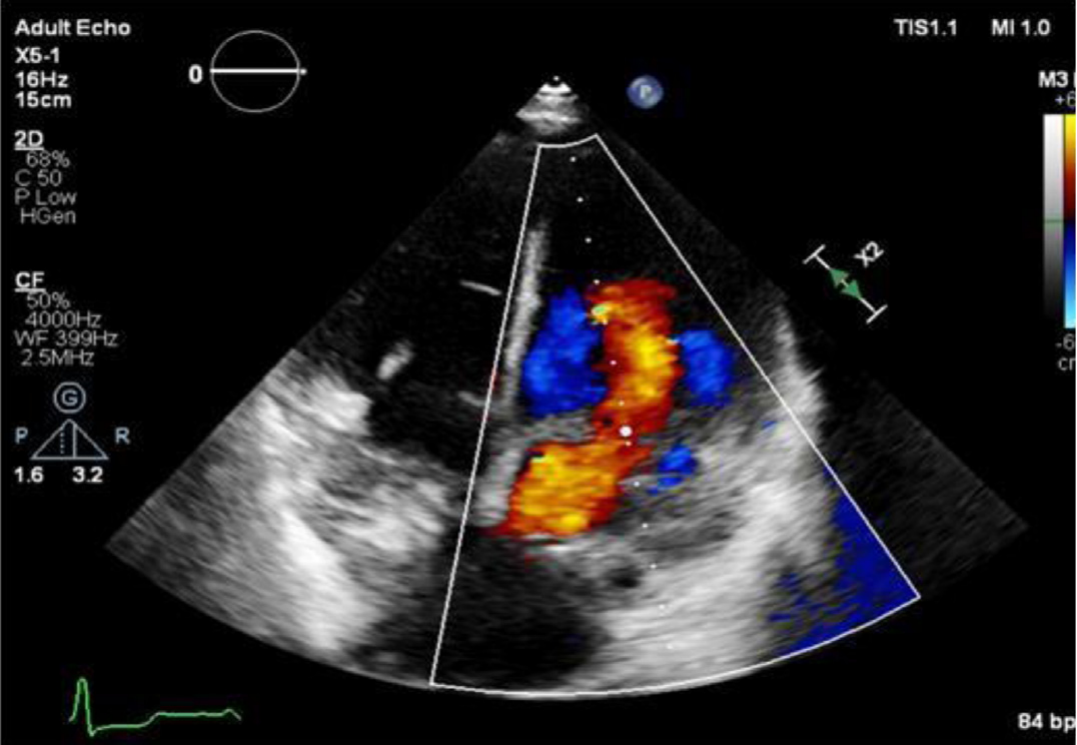
Transthoracic Echocardiography Apical 4-chamber view postoperatively shows the flow across the left atrium with almost no aliasing.

Under general anesthesia, the patient underwent minimally invasive cardiac surgery via a right anterolateral thoracotomy. Heparin was administered, and cannulation of the right common femoral vein and artery was performed. After aortic cross clamping, antegrade cardioplegia was delivered, and cardiac arrest was achieved. A left atriotomy was performed, and the membranous portion of the cor triatriatum was recognized and resected (Figure 6) along with a portion of the muscular septation. A mitral valve water test demonstrated proper coaptation with no evidence of regurgitation. The heart was de-aired, the aortic cross-clamp was removed, and the heart resumed spontaneous sinus rhythm. Intraoperative transesophageal echocardiography confirmed good biventricular function, mild mitral regurgitation, no residual tricuspid regurgitation, and a reduced mean gradient of 4 mm Hg across the left atrium (Figure 7). The patient was transferred to the intensive care unit, intubated, and sedated. She was extubated 6 hours postprocedure and discharged home on day 5 postoperatively with no complications.

## Discussion

CTS is a rare and a complex congenital heart disease, and it can be associated with other congenital cardiac anomalies, like in our patient, with persistent left SVC to coronary sinus. Its presentation varies widely in symptoms and age, and it causes symptoms that mimic mitral stenosis. The time of presentation depends on severity of obstruction that probably increases with time.

Surgical intervention is the definitive treatment for such a lesion, with resection of the fibromuscular membrane. The usual approach is midline sternotomy with complete repair.

**Figure 8:**
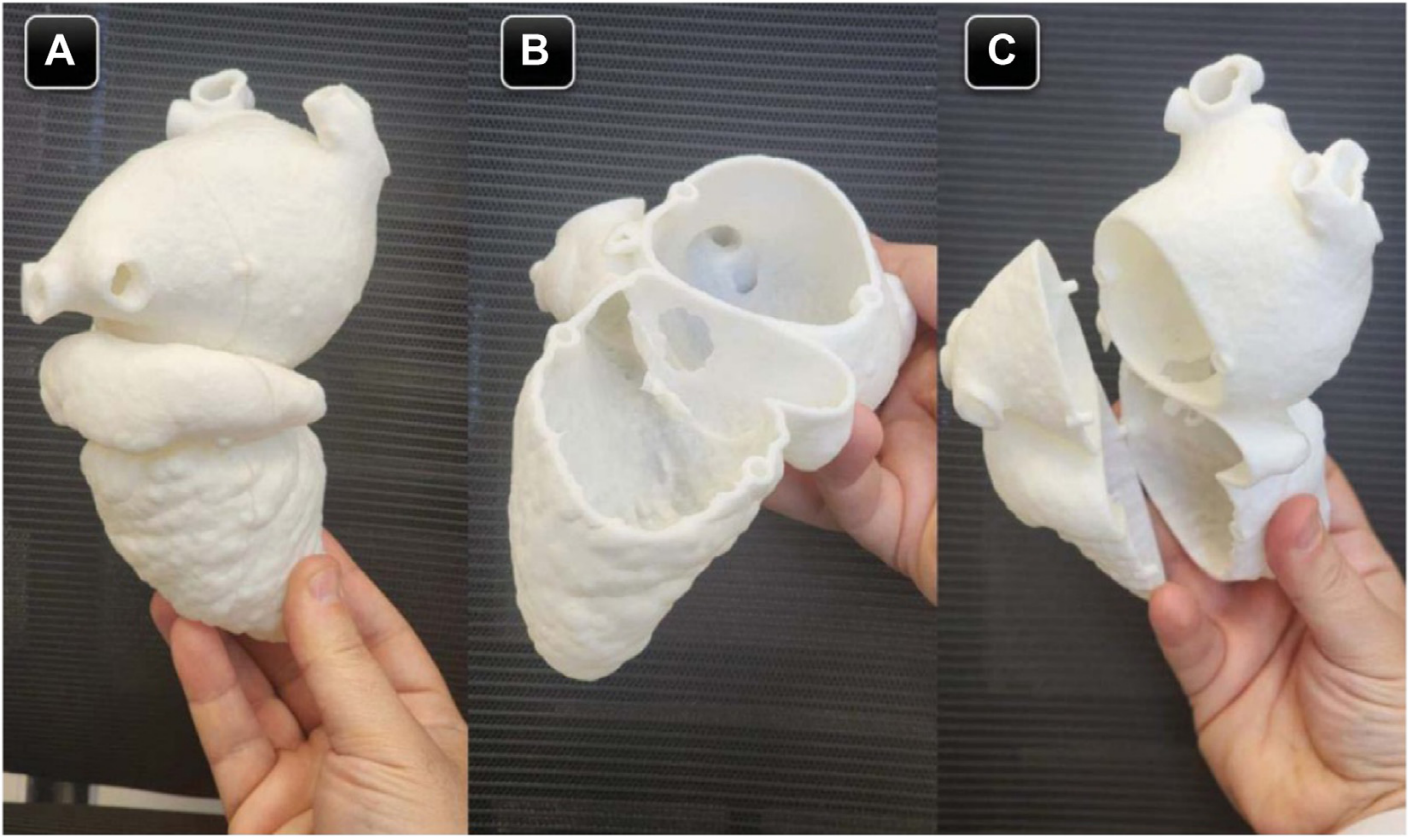
Three-Dimensional Printed Model of the Heart in a Normal Background Pictures of the 3D model in different projections; show the left atrium and ventricle posterior view (A), the model opened and shows the membrane and the hole in it (B and C).

The advancement and advantages of a minimally invasive surgical approach with thoracotomy include fast recovery time, less pain, and fewer scar marks, making it a preferable approach, especially in a young teenager. However, the lack of expertise in repairing such a rare complex lesion via the limited surgical view allowed by the minimally invasive approach, the distorted anatomy due to severely dilated left atrium with a false proximal cavity, and the persistent left SVC to coronary sinus initiated the idea of performing a 3D printing model of the heart based on the computed tomography scan images (Figures 4 and 8), and allowing the surgeon to do virtual surgical planning for the operation.

Simulation of surgery, combining the 3D printed model slicing and computed tomography scan images, allowed the surgeon deciding the resection direction and the diameter of the circle/hole he needed to create in the fibromuscular membrane to open the drainage of the pulmonary veins without injuries to nearby structures like the coronary sinus, mitral valve, or interatrial septum.

The patient's heart had severely dilated left atrium that had distorted the normal anatomy, and the pulmonary veins and the coronary sinus were compressed and displaced. Therefore, the 3D virtual model was used via a video call with the technician and cardiologist by the surgeon, who led a surgical simulation, combining the 3D model cutting images with the computed tomography scan to decide where to cut in the left atrium to avoid any injury to pulmonary veins and not create an iatrogenic atrial septal defect.

## Conclusions

Minimally invasive thoracotomy is a safe approach and can be used to mend some hearts with complex congenital defects. 3D printing of hearts with congenital heart diseases and combining it with other modalities of imaging, like echocardiography and CT scan, may allow better understanding of the anatomy and the luxury of doing virtual surgeries in planning for the real operations.

## Funding Support and Author Disclosures

The authors have reported that they have no relationships relevant to the contents of this paper to disclose.

## References

[1] Jha A.K., Makhija N. (2017). Cor triatriatum: a review. Semin Cardiothorac Vasc Anesth.

[2] Niwayama G. (1960). Cor triatriatum. Am Heart J.

[3] Sankhyan L.K., Anderson R.H., Chowdhury U.K. (2021). Surgical management of divided atrial chambers. J Card Surg.

[4] Jegier W., Gibbons J.E., Wiglesworth F.W. (1963). Cor triatriatum: clinical, hemodynamic and pathological studies surgical correction in early life. Pediatrics.

[5] Bezante G.P., Deferrari L., Molinari G., Valbusa A., Rosa G., Barsotti A. (2002). Cor triatriatum sinistrum and persistent left superior vena cava: an original association. Eur J Echocardiogr.

[6] Humpl T., Reineker K., Manlhiot C., Dipchand A.I., Coles J.G., McCrindle B.W. (2010). Cor triatriatum sinistrum in childhood. A single institution's experience. Can J Cardiol.

[7] Eichholz J.L., Hodroge S.S., Crook J.J., Mack J.W., Wortham D.C. (2013). Cor triatriatum sinister in a 43-year-old man with syncope. Tex Heart Inst J.

[8] Mendez A.B., Colchero T., Garcia-Picart J., Vila M., Subirana M.T., Sionis A. (2013). Unusual case of new-onset heart failure due to cor triatriatum sinister. Eur J Heart Fail.

[9] Walling S., Palma R., Chubet L. (2013). Combined 2D and 3 D transthoracic and transesophageal echocardiography in evaluation of nonobstructive and obstructive cor triatriatum sinister. J Diagn Med Sonogr.

[10] Lupinski R.W., Shankar S., Wong K.Y. (2001). Cor triatriatum: clinical presentation of 18 cases. Asian Cardiovasc Thorac Ann.

